# Is a Cutoff of 10% Appropriate for the Change-in-Estimate Criterion of Confounder Identification?

**DOI:** 10.2188/jea.JE20130062

**Published:** 2014-03-05

**Authors:** Paul H. Lee

**Affiliations:** 1 School of Public Health, University of Hong Kong, Hong Kong; 2 School of Nursing, Hong Kong Polytechnic University, Hong Kong

**Keywords:** causality, confounding factors, regression, simulation, statistical models

## Abstract

**Background:**

When using the change-in-estimate criterion, a cutoff of 10% is commonly used to identify confounders. However, the appropriateness of this cutoff has never been evaluated. This study investigated cutoffs required under different conditions.

**Methods:**

Four simulations were performed to select cutoffs that achieved a significance level of 5% and a power of 80%, using linear regression and logistic regression. A total of 10 000 simulations were run to obtain the percentage differences of the 4 fitted regression coefficients (with and without adjustment).

**Results:**

In linear regression, larger effect size, larger sample size, and lower standard deviation of the error term led to a lower cutoff point at a 5% significance level. In contrast, larger effect size and a lower exposure–confounder correlation led to a lower cutoff point at 80% power. In logistic regression, a lower odds ratio and larger sample size led to a lower cutoff point at a 5% significance level, while a lower odds ratio, larger sample size, and lower exposure–confounder correlation yielded a lower cutoff point at 80% power.

**Conclusions:**

Cutoff points for the change-in-estimate criterion varied according to the effect size of the exposure–outcome relationship, sample size, standard deviation of the regression error, and exposure–confounder correlation.

## INTRODUCTION

Confounders are defined as variables that distort the true effect between exposure and outcome.^1^ Specifically, confounders are variables that are associated with both exposure and outcome but not affected by either the exposure or outcome.^2^ Identification of confounders is important in observational studies of the effect of an exposure on an outcome, as confounders bias estimates of the true causal effect. There are many strategies to identify confounders, eg, forward, backward, and stepwise variable selection.^3^ Among these strategies, simulation studies have shown that the best is the change-in-estimate criterion,^4^^,^^5^ in which confounders are defined as variables that alter the unadjusted exposure–outcome effect by a certain percentage. A cutoff of 10% is commonly cited in the literature.^1^

There are very few studies of the statistical properties of the change-in-estimate criterion.^1^ In particular, the appropriateness of the 10% cutoff point has never been evaluated. It is very likely that the exposure–outcome relationship, sample size, standard deviation (SD) of the regression error, and exposure–confounder correlation affect the cutoff point. This pioneer study attempts to answer the question, “What are the factors associated with the change-in-estimate cutoff point?”. Using a simulation technique, I determine the required cutoffs to achieve a significance level (or type I error) of 5% and a power (1 − [type II error]) of 80%, under different conditions of exposure–outcome relationship, sample size, SD of the regression error, and exposure–confounder correlation.

## METHODS

Four simulations were carried out to identify a cutoff for the change-in-estimate criterion that achieves a significance level of 5% and a power of 80%. Throughout this article, *X*, *Y*, and *Z* will be used to denote exposure, outcome, and possible confounder, respectively. The first simulation mimicked a situation in which *Z* is not a true confounder of the relationship between *X* and *Y*. The simulated data were drawn from the model *Y* = effect_size * *X* + SD(*error*) * *error*, where *X* and error followed a standard normal distribution. The standard normal variable *Z* was independently simulated. The second simulation mimicked a situation in which *Z* is a true confounder of the relationship between *X* and *Y*. The simulated data of the second simulation were drawn from the model *Y* = effect_size * *X* + *Z* + SD(*error*) * *error*, where *X*, *Z*, and the error followed a standard normal distribution. By definition, a confounder is associated with the exposure; therefore, *X* and *Z* were drawn such that they were correlated with specific Spearman correlations. For both simulations, 2 linear regressions were fitted: one treated *Y* as the dependent variable and *X* as the independent variable and the other linear regression further adjusted for *Z*. The percentage differences of the 2 fitted regression coefficients (the absolute value of the difference between the adjusted coefficient and the crude coefficient divided by the crude coefficient) from 10 000 simulation runs were obtained. The 95th and 20th percentiles of these percentage differences were used as the cutoff for a significance level of 5% and power of 80%, respectively. The third and fourth simulations were similar to the first and second simulations but were based on logistic regression. The binary outcome *Y* of the third and fourth simulations was drawn from the models *Prob*(*Y* = 1) = *ln*(odds ratio) * *X* + *error* and *Prob*(*Y* = 1) = *ln*(odds ratio) * *X* + *Z* + *error*, respectively, where error followed a standard logistic distribution. To compare the performance of the cutoffs obtained by the aforementioned simulations with that of the commonly used 10% cutoff, additional simulation studies were conducted in order to compute the root-mean-square error (RMSE) of the effect estimators obtained. RMSE equals $\sum_{}^{}{}_{i-1}^{k}\frac{\sqrt{{\left(\hat{\beta }-\beta \right)}^{2}}}{k}$ where *k*, $\hat{\beta }$, and *β* are the simulation size, estimated effect of exposure, and true effect of exposure, respectively. For simplicity, only the case in which the obtained cutoff deviated most from the simulation with the 10% cutoff was simulated 10 000 times.

In both simulations, different levels of effect size (linear regression: 0.1, 0.2, 0.3, 0.4, 0.5; logistic regression, odds ratio [OR]: 1.5, 2, 2.5, 3, 3.5), SD of the error term (0.1, 0.2, 0.3, 0.4), and sample size (500, 1000, 5000, 10 000) were tested. The effect sizes are in the range of a small-to-medium effect in both linear regression^6^ and logistic regression.^7^ For the second simulation, different degrees of correlation between *X* and *Z* (0.1, 0.2, 0.3, 0.4) were also tested.

Finally, to demonstrate the use of this proposed method in identifying confounders to be adjusted, an example of linear regression of the association between physical activity and lung function using the publicly available National Health and Nutrition Examination Survey (NHANES) 2009–2010 data will be presented. The details of the survey are available at the official website (http://wwwn.cdc.gov/nchs/nhanes/search/nhanes09_10.aspx). All simulations were carried out using R version 2.15.0.

## RESULTS

Table 1 shows the results of the first simulation. Larger effect size, larger sample size, and smaller SD of the error term led to lower cutoff point at a 5% significance level. These factors had a strong effect on the cutoff. The cutoff points for an effect size of 0.1 were 5.13 times (sample size = 10 000; SD(error) = 1) to 13.93 times (sample size = 500; SD(error) = 2) those for an effect size of 0.5. The cutoff points for a sample size of 500 were 19.71 times (effect size = 0.5; SD(error) = 1) to 52.27 times (effect size = 0.2; SD(error) = 4) those for a sample size of 10 000. The cutoff points for an SD of 4 were 3.84 times (sample size = 10 000; effect size = 0.4) to 10.35 times (sample size = 500; effect size = 0.2) those for an SD of 1.

**Table 1.** The 95th percentile of the percentage difference in estimates of the effect of *X* with and without adjustment for a randomly generated variable, *Z* (linear regression, simulation size = 10 000)

	SD (Error)	Effect size of *X*				

		0.1	0.2	0.3	0.4	0.5
Sample size = 500	1	6.85%	2.38%	1.53%	1.12%	0.87%
	2	25.20%	6.86%	3.32%	2.37%	1.81%
	3	34.62%	14.32%	6.54%	3.98%	3.03%
	4	38.79%	24.60%	12.01%	7.01%	4.48%
Sample size = 1000	1	2.54%	1.13%	0.75%	0.56%	0.43%
	2	10.49%	2.61%	1.57%	1.12%	0.89%
	3	18.11%	5.21%	2.63%	1.77%	1.36%
	4	24.03%	10.20%	4.20%	2.59%	1.99%
Sample size = 5000	1	0.46%	0.23%	0.15%	0.11%	0.09%
	2	0.98%	0.44%	0.30%	0.22%	0.17%
	3	2.03%	0.72%	0.45%	0.34%	0.26%
	4	3.49%	1.03%	0.61%	0.44%	0.35%
Sample size = 10 000	1	0.23%	0.11%	0.07%	0.06%	0.04%
	2	0.48%	0.22%	0.14%	0.11%	0.09%
	3	0.76%	0.35%	0.22%	0.16%	0.13%
	4	1.23%	0.47%	0.30%	0.21%	0.18%

The performance of the new proposed cutoff criterion and the 10% change-in-estimate criterion were evaluated using the cutoff point obtained in the simulation that deviated most from the 10%, that is, sample size equals 500, SD (Error) equals 4, and effect size of *X* equals 0.1. The proposed cutoff was 38.79%. In 10 000 simulation runs, 1309 runs yielded change-in-estimate values between 10% and 38.79%. Among these simulations, the RMSE was 1.31%, using the proposed cutoff, which was smaller than that of the 10% cutoff (RMSE = 1.33%).

Table 2 shows the results of the second simulation. Larger effect size and a lower exposure–confounder correlation led to a lower cutoff point at 80% power. The cutoff points for an effect size of 0.1 were 1.67 times (sample size = 500; SD(error) = 4; correlation = 0.4) to 13.93 times (sample size = 500; SD(error) = 1; correlation = 0.1) those for an effect size of 0.5.

**Table 2.** The 20th percentile of the percentage difference in estimates of the effect of *X* with and without adjustment for a confounder, *Z* (linear regression, simulation size = 10 000)

		SD (Error)	Effect size of *X*				

			0.1	0.2	0.3	0.4	0.5
Cor(*X*, *Z*) = 0.1	Sample size = 500	1	36.06%	23.44%	17.28%	13.28%	10.89%
		2	32.29%	22.04%	16.35%	12.97%	10.87%
		3	27.88%	19.83%	15.39%	12.25%	10.50%
		4	23.94%	18.46%	14.54%	11.77%	10.00%
	Sample size = 1000	1	40.09%	26.44%	19.44%	15.32%	12.73%
		2	36.86%	24.97%	19.03%	15.13%	12.46%
		3	33.31%	23.79%	18.17%	14.68%	12.18%
		4	30.36%	21.94%	17.23%	14.06%	11.92%
	Sample size = 5000	1	45.67%	30.26%	22.59%	18.00%	14.94%
		2	43.66%	29.65%	22.27%	17.84%	14.86%
		3	41.65%	28.62%	21.82%	17.59%	14.65%
		4	39.41%	27.88%	21.30%	17.29%	14.43%
	Sample size = 10 000	1	46.99%	31.23%	23.29%	18.60%	15.48%
		2	45.69%	30.70%	23.05%	18.42%	15.41%
		3	43.82%	30.12%	22.71%	18.31%	15.26%
		4	42.33%	29.29%	22.36%	18.06%	15.08%
Cor(*X*, *Z*) = 0.2	Sample size = 500	1	57.57%	43.02%	34.30%	28.46%	24.26%
		2	51.42%	39.62%	32.39%	27.14%	23.35%
		3	46.15%	36.66%	30.13%	25.52%	22.23%
		4	42.22%	33.93%	28.33%	23.89%	20.95%
	Sample size = 1000	1	60.08%	45.09%	35.95%	29.93%	25.52%
		2	55.54%	42.80%	34.53%	28.96%	24.80%
		3	50.99%	40.20%	33.11%	27.87%	23.93%
		4	47.33%	37.71%	31.20%	26.54%	22.93%
	Sample size = 5000	1	63.72%	47.82%	38.24%	31.81%	27.22%
		2	61.23%	46.57%	37.47%	31.32%	26.92%
		3	58.87%	45.18%	36.77%	30.69%	26.45%
		4	57.17%	43.91%	35.78%	30.12%	26.01%
	Sample size = 10 000	1	64.53%	48.46%	38.72%	32.27%	27.60%
		2	62.93%	47.56%	38.27%	31.90%	27.42%
		3	61.18%	46.64%	37.61%	31.49%	27.08%
		4	59.15%	45.58%	36.98%	31.13%	26.73%
Cor(*X*, *Z*) = 0.3	Sample size = 500	1	67.22%	54.19%	45.20%	38.72%	33.78%
		2	61.45%	50.41%	42.51%	36.67%	32.28%
		3	56.37%	46.84%	39.73%	34.38%	30.52%
		4	51.22%	43.37%	37.28%	32.41%	28.81%
	Sample size = 1000	1	69.52%	55.88%	46.61%	39.86%	34.91%
		2	65.44%	52.99%	44.68%	38.42%	33.71%
		3	61.13%	50.26%	42.57%	36.95%	32.53%
		4	57.18%	47.61%	40.42%	35.21%	31.19%
	Sample size = 5000	1	72.44%	58.17%	48.54%	41.55%	36.31%
		2	70.41%	56.76%	47.55%	40.89%	35.84%
		3	68.35%	55.31%	46.48%	40.03%	35.20%
		4	66.46%	53.84%	45.43%	39.26%	34.59%
	Sample size = 10 000	1	73.22%	58.68%	48.95%	41.93%	36.68%
		2	71.70%	57.67%	48.25%	41.44%	36.34%
		3	70.11%	56.62%	47.47%	40.82%	35.87%
		4	68.69%	55.60%	46.77%	40.25%	35.44%
Cor(*X*, *Z*) = 0.4	Sample size = 500	1	73.14%	61.27%	52.67%	46.15%	41.01%
		2	67.84%	57.54%	57.54%	49.61%	38.98%
		3	62.42%	53.35%	46.36%	41.05%	36.87%
		4	58.00%	49.47%	43.77%	38.49%	34.72%
	Sample size = 1000	1	75.21%	62.93%	54.00%	47.39%	42.01%
		2	70.99%	59.89%	51.80%	45.46%	40.45%
		3	66.92%	56.77%	49.28%	43.57%	38.97%
		4	63.89%	54.51%	47.34%	42.09%	37.42%
	Sample size = 5000	1	77.81%	64.99%	55.71%	48.79%	43.38%
		2	75.71%	63.57%	54.72%	47.99%	42.67%
		3	73.93%	62.00%	53.58%	46.98%	41.90%
		4	71.88%	60.73%	52.54%	46.13%	41.09%
	Sample size = 10 000	1	78.47%	65.50%	56.12%	49.15%	43.71%
		2	77.02%	64.45%	55.39%	48.57%	43.24%
		3	75.58%	63.44%	54.59%	47.89%	42.67%
		4	74.32%	62.45%	53.79%	47.19%	42.16%

Table 3 shows the results of the third simulation. A lower OR and larger sample size led to a smaller cutoff point at a 5% significance level. The OR had a weak effect on cutoff values, but sample size had a strong effect on the cutoff. The cutoff points for an OR of 1.5 were 1.53 times (sample size = 10 000) to 1.68 times (sample size = 1000) those for an OR of 3.5. The cutoff points for a sample size of 500 were 19.97 times (OR = 2) to 21.86 times (OR = 3.5) those for a sample size of 10 000.

**Table 3.** The 95th percentile of the percentage difference in estimates of the effect of *X* with and without adjustment for a randomly generated variable, *Z* (logistic regression, simulation size = 10 000)

Sample size	Odds ratio of *X*				

	1.5	2.0	2.5	3.0	3.5
500	0.99%	1.09%	1.30%	1.39%	1.60%
1000	0.47%	0.54%	0.63%	0.70%	0.79%
5000	0.09%	0.11%	0.12%	0.14%	0.15%
10 000	0.05%	0.05%	0.06%	0.07%	0.07%

Table 4 shows the results of the fourth simulation. A lower OR, larger sample size, and lower exposure–confounder correlation led to a lower cutoff point at 80% power. All had a weak effect on cutoff values. The cutoff points for an OR of 1.5 were 1.08 times (sample size = 1000; correlation = 0.1) to 1.16 times (sample size = 10 000; correlation = 0.4) those for an OR of 3.5. The cutoff points for a sample size of 500 were 4.31 times (OR = 3; correlation = 0.2) to 4.66 times (OR = 1.5; correlation = 0.1) those for a sample size of 10 000. The cutoff points for a correlation of 0.1 were 4.31 times (OR = 3; correlation = 0.2) to 4.66 times (OR = 1.5; correlation = 0.1) those for a correlation of 0.4.

**Table 4.** The 20th percentile of the percentage difference in estimates of the effect of *X* with and without adjustment for a confounder, *Z* (logistic regression, simulation size = 10 000)

	Sample size	Odds ratio of *X*				

		1.5	2.0	2.5	3.0	3.5
Cor(*X*, *Z*) = 0.1	500	1.21%	1.25%	1.27%	1.30%	1.32%
	1000	0.85%	0.85%	0.87%	0.89%	0.91%
	5000	0.37%	0.38%	0.39%	0.40%	0.41%
	10 000	0.26%	0.27%	0.28%	0.28%	0.29%
Cor(*X*, *Z*) = 0.2	500	2.41%	2.46%	2.50%	2.56%	2.69%
	1000	1.69%	1.74%	1.76%	1.83%	1.88%
	5000	0.76%	0.78%	0.80%	0.83%	0.85%
	10 000	0.54%	0.56%	0.57%	0.59%	0.59%
Cor(*X*, *Z*) = 0.3	500	3.79%	3.90%	3.95%	4.07%	4.17%
	1000	2.58%	2.64%	2.78%	2.86%	2.88%
	5000	1.16%	1.21%	1.23%	1.29%	1.31%
	10 000	0.83%	0.95%	0.89%	0.91%	0.91%
Cor(*X*, *Z*) = 0.4	500	5.12%	5.26%	5.47%	5.61%	5.74%
	1000	3.64%	3.72%	3.82%	3.93%	4.03%
	5000	1.61%	1.68%	1.74%	1.79%	1.81%
	10 000	1.14%	1.19%	1.23%	1.24%	1.32%

To illustrate the present method, a linear regression was fitted to the NHANES 2009–2010 dataset to examine the association of adequate physical activity (ie, ≥150 minutes of moderate-to-vigorous physical activity per week^8^) with lung function (using forced expiratory volume in 1 second, FEV_1_, as a proxy). Only participants aged 20 years or older who provided high-quality spirometry data were included, and the current sample consisted of 4611 participants. Using the R code provided in the Appendix, it was found that a cutoff of 0.18% achieved a significance level of 5%. In examining the list of potential confounders^9^^–^^11^ (age, sex, ethnicity, education, marital status, body mass index, smoking, history of stroke, history of heart attack), the change in the estimate was larger than 0.18% for all variables except smoking (0.16%). The raw and adjusted associations between adequate physical activity and FEV_1_ were 458.33 (SE 25.46) and 78.95 (SE 16.63), respectively. As a reference, using the 10% cutoff point, only age (33.8%), sex (31.5%), and marital status (13.4%) required adjustment; the association was 142.26 (SE 17.45).

## DISCUSSION

Because the change-in-estimate criterion was shown to be best^4^^,^^5^ at identifying confounders, it became the most popular strategy among the many used for confounder selection. Those adopting the change-in-estimate algorithm usually used a single cutoff, regardless of the characteristics of the dataset. However, the present simulation study showed that cutoff points for the change-in-estimate criterion vary according to the effect size of the exposure–outcome relationship, sample size, SD of the regression error, and exposure–confounder correlation.

The 10% cutoff is the most commonly used indicator of a confounding effect. However, this simulation study shows that varying cutoff values should be used with different settings. Furthermore, although the 10% cutoff criterion yielded a power of at least 80% in all simulated scenarios, the significance level sometimes decreased to less than 5%. For example, in the scenario with a sample size of 500, a SD of the error term of 4, and an effect size of 0.1, a cutoff of 38.79% was required to achieve a significance level of 5%. Additional simulations showed that this cutoff performed better than the commonly used 10% cutoff.

To consider whether a possible confounder should be adjusted, the following approach should be used. First, simulate a random variable that follows a standard normal distribution. Second, fit a linear regression on the standardized outcome by the standardized exposure. Third, compute the percentage difference of the regression slope, with and without adjusting for the random variable, and obtain the 95th percentile. Lastly, use this 95th percentile as the cutoff for the change-in-estimate criterion, that is, variables that induce a change greater than this 95th percentile will be treated as confounders. This procedure was demonstrated using the NHANES 2009–2010 data, and the relevant R code is included in the Appendix. The power of this change-in-estimate criterion can also be computed by simulation.

Note that the change-in-estimate criterion and other data-driven strategies for confounder identification can only suggest the possible confounding effect of a variable; they cannot identify the causal effect of the confounder on the outcome. Therefore, in adjusting for possible confounders, one must note that these adjusted confounders are neither the cause of the exposure nor the cause of the outcome.^12^^,^^13^ Before automated confounder identification, researchers were recommended to select theoretically possible confounders by using directed acyclic graphs.^14^

This simulation study focused on continuous and binary outcomes. Further studies of the change-in-estimate criterion for ordinal and survival outcomes are warranted and can be performed after slight modification of the R code provided in the Appendix.

## APPENDIX

R code for the first simulation:
sim_size <- 10000 ## Simulation size
sample_size <- 1000 ## Sample size of each simulation
effect <- 0.1 ## Effect size
var_e <- 2 ## Variance of error
estimate <- 1:sim_size
## Simulation starts
for (i in 1:sim_size){
x <- rnorm(sample_size)
z <- rnorm(sample_size)
e <- rnorm(sample_size)*var_e
y <- effect*x + e
reg <- lm(y~x)
test1 <- reg$coefficients[2]
reg <- lm(y~x+z)
test2 <- reg$coefficients[2]
ratio <- test2/test1
if (ratio<1) ratio = 2-ratio
estimate[i] <- ratio
}
## Simulation ends
quantile(estimate,0.95) ## Output the 95% cutoff
R code for the second simulation:
sim_size <- 10000 ## Simulation size
sample_size <- 500 ## Sample size of each simulation
effect <- 0.1 ## Effect size
var_e <- 1 ## Variance of error
cor_x_z <- 0.1 ## Exposure-confounder correlation
estimate <- 1:sim_size
## Simulation starts
for (i in 1:sim_size){
x <- rnorm(sample_size)
z <- cor_x_z*x+sqrt(1-cor_x_z*cor_x_z)*rnorm(sample_size)
e <- rnorm(sample_size)*var_e
y <- effect*x + z + e
reg <- lm(y~x)
test1 <- reg$coefficients[2]
reg <- lm(y~x+z)
test2 <- reg$coefficients[2]
ratio <- test2/test1
if (ratio<1) ratio = 2-ratio
estimate[i] <- ratio
}
## Simulation ends
quantile(estimate,0.8) ## Output the 95% cutoff
R code for the third simulation:
sim_size <- 10000 ## Simulation size
sample_size <- 5000 ## Sample size of each simulation
OR <- 3.5 ## Odds ratio
estimate <- 1:sim_size
## Simulation starts
for (i in 1:sim_size){
x <- rnorm(sample_size)
z <- rnorm(sample_size)
p <- exp(log(OR)*x) / (1+exp(log(OR)*x))
y <- 1:sample_size
for (j in 1:sample_size){
y[j] <- sample(0:1,1,rep=TRUE,prob=c(1-p[j],p[j]))
}
reg <- glm(y~x, family = binomial)
test1 <- exp(reg$coefficients[2])
reg <- glm(y~x+z, family = binomial)
test2 <- exp(reg$coefficients[2])
ratio <- test2/test1
if (ratio<1) ratio = 2-ratio
estimate[i] <- ratio
}
## Simulation ends
quantile(estimate,0.95) ## Output the 95% cutoff
R code for the fourth simulation:
sim_size <- 10000 ## Simulation size
sample_size <- 10000 ## Sample size of each simulation
OR <- 1.5 ## Odds ratio
cor_x_z <- 0.4 ## Exposure-confounder correlation
estimate <- 1:sim_size
## Simulation starts
for (i in 1:sim_size){
x <- rnorm(sample_size)
z <- cor_x_z*x+sqrt(1-cor_x_z*cor_x_z)*rnorm(sample_size)
p <- exp(log(OR)*x) / (1+exp(log(OR)*x))
y <- 1:sample_size
for (j in 1:sample_size){
y[j] <- sample(0:1,1,rep=TRUE,prob=c(1-p[j],p[j]))
}
reg <- glm(y~x, family = binomial)
test1 <- exp(reg$coefficients[2])
reg <- glm(y~x+z, family = binomial)
test2 <- exp(reg$coefficients[2])
ratio <- test2/test1
if (ratio<1) ratio = 2-ratio
estimate[i] <- ratio
}
## Simulation ends
quantile(estimate,0.8) ## Output the 95% cutoff
R code for the real example:
SPX_PA <- read.csv("C:/SPX_PA.csv",header=T) ## read the data
sim_size <- 10000
estimate <- 1:sim_size
## simulation starts
for (i in 1:sim_size){
x <- SPX_PA[,1]/sd(SPX_PA[,1])
z <- rnorm(nrow(SPX_PA))
e <- rnorm(nrow(SPX_PA))
y <- SPX_PA[,2]/sd(SPX_PA[,2])
reg <- lm(y~x)
test1 <- reg$coefficients[2]
reg <- lm(y~x+z)
test2 <- reg$coefficients[2]
ratio <- test2/test1
if (ratio<1) ratio = 2-ratio
estimate[i] <- ratio
}
quantile(estimate,0.95) ## cutoff = 0.18%
